# Supplementary material for: Predicting antimicrobial resistance using conserved genes
Source: PLoS Comput Biol. 2020 Oct 19;16(10):e1008319. doi: 10.1371/journal.pcbi.1008319 (PMC7595632; doi:10.1371/journal.pcbi.1008319)
Supplement: S1 File — (PDF) [file pcbi.1008319.s001.pdf]

Supplementary Figures For:

Predicting antimicrobial resistance using conserved genes

Marcus Nguyen, Robert Olson, Maulik Shukla, Margo VanOeffelen, and James J. Davis

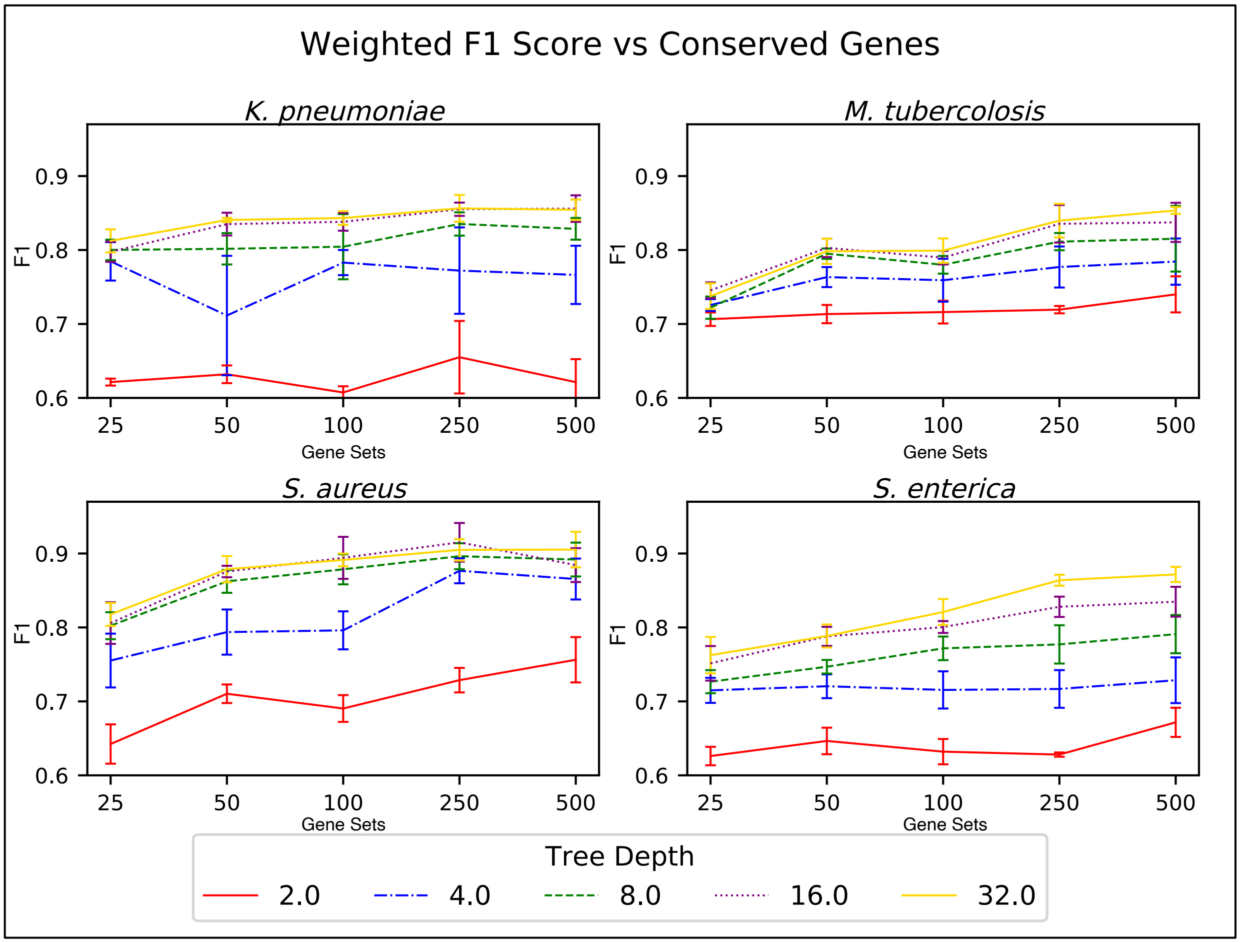

Figure A. The effect of tree depth on models built from core gene sets of various sizes. K-mer-based XGB models were built for randomly-selected core gene sets ranging in size from 25-500 genes. The X-axis depicts the core gene set size, and the Y-axis depicts F1 scores. Each line represents models built at varying tree depths from 2-32. Error bars are the 95% confidence interval.

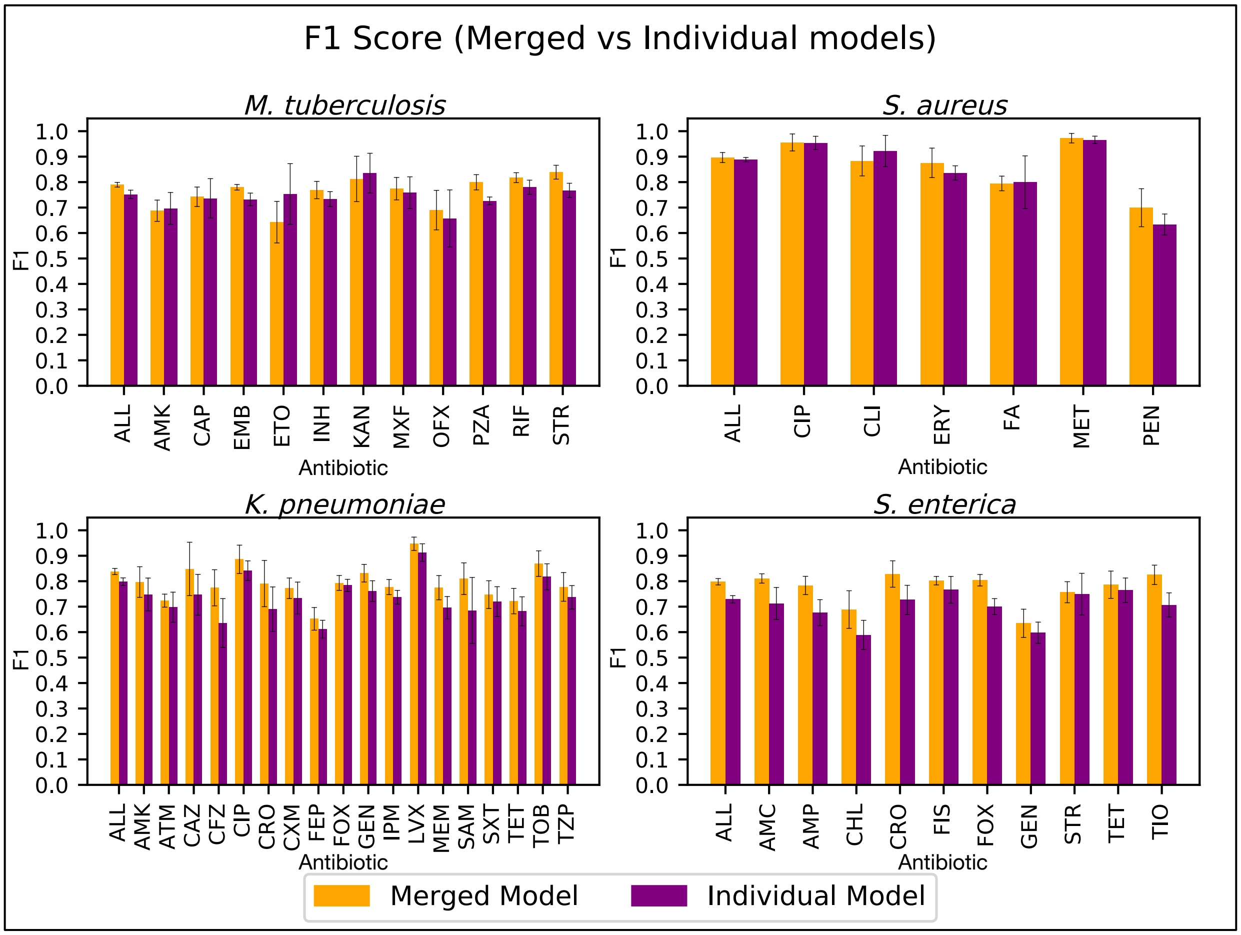

Figure B. A comparison of F1 scores for merged models, where all antibiotics are included in a single model, and individual models built for each antibiotic. K-mer-based XGB models were built from the same set of 100 randomly selected core genes. Error bars represent the 95% confidence intervals. Antibiotic abbreviations are defined in Table 2.

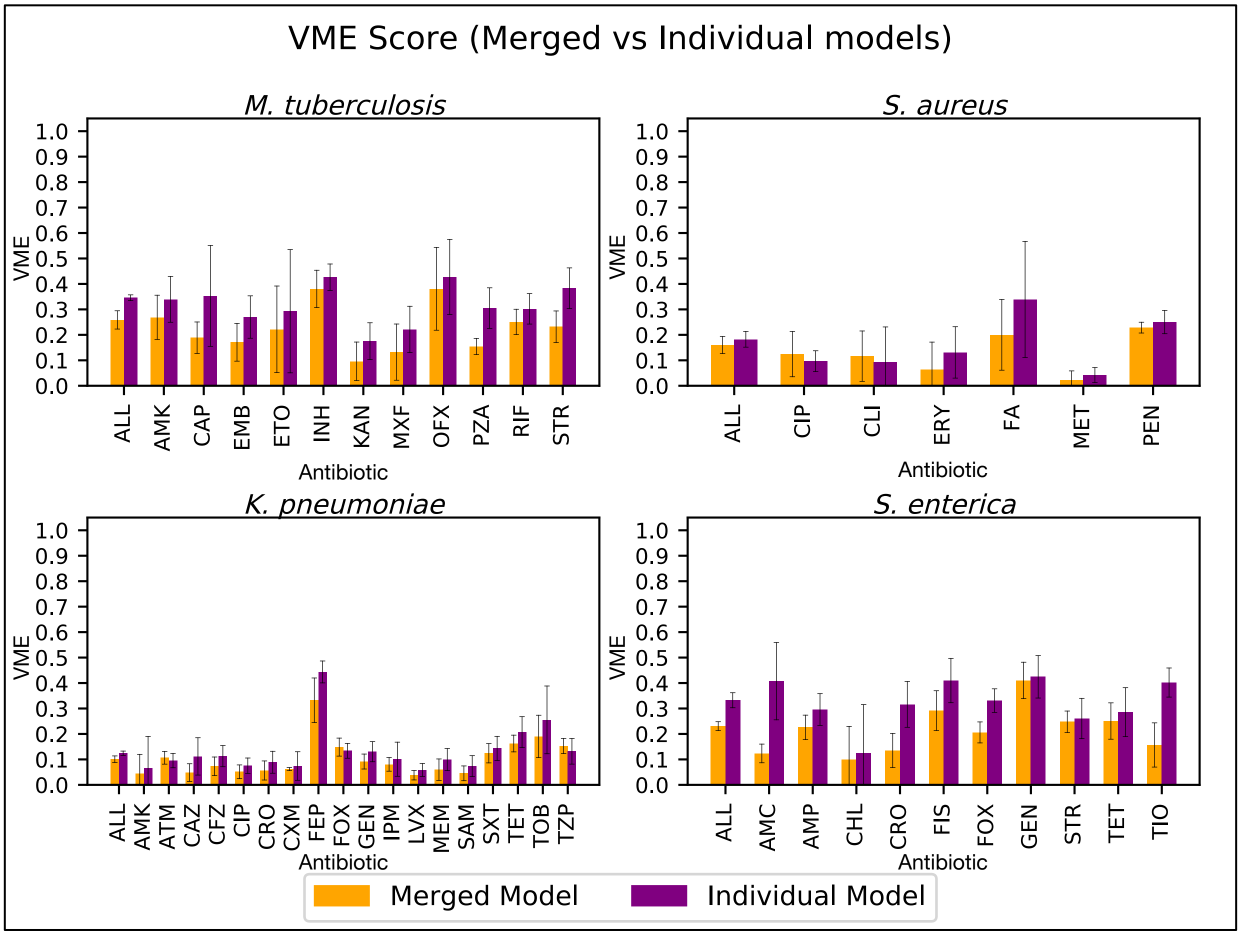

Figure C. A comparison of very major error rates for merged models, where all antibiotics are included in a single model, and individual models built for each antibiotic. K-mer-based XGB models were built from the same set of 100 randomly selected core genes. Very major errors are defined as resistant genomes that are misclassified as being susceptible. Error bars represent the 95% confidence intervals. Antibiotic abbreviations are defined in Table 2.

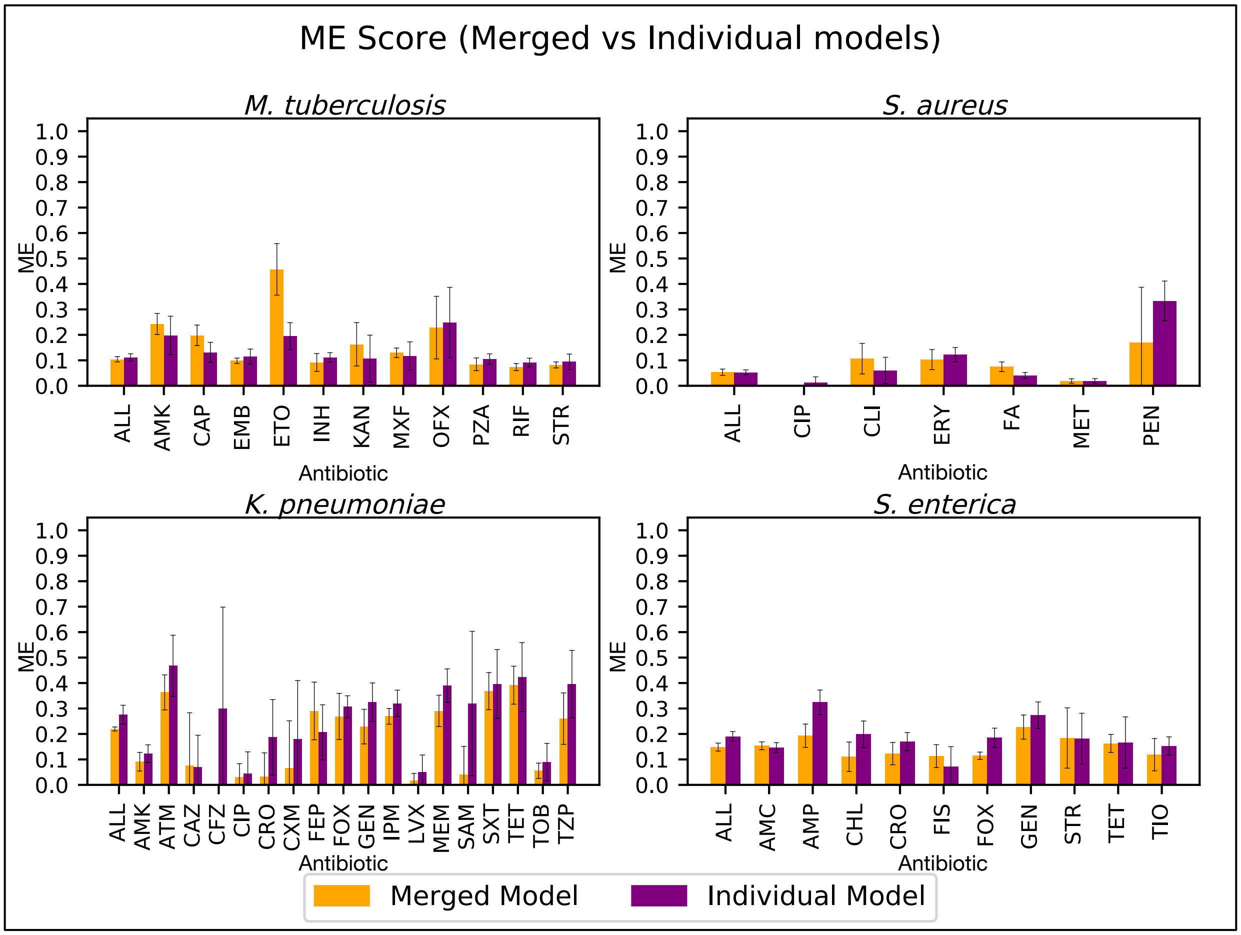

Figure D. A comparison of major error rates for merged models, where all antibiotics are included in a single model, and individual models built for each antibiotic. K-mer-based XGB models were built from the same set of 100 randomly selected core genes. Major errors are defined as susceptible genomes that are misclassified as being resistant. Error bars represent the 95% confidence intervals. Antibiotic abbreviations are defined in Table 2.

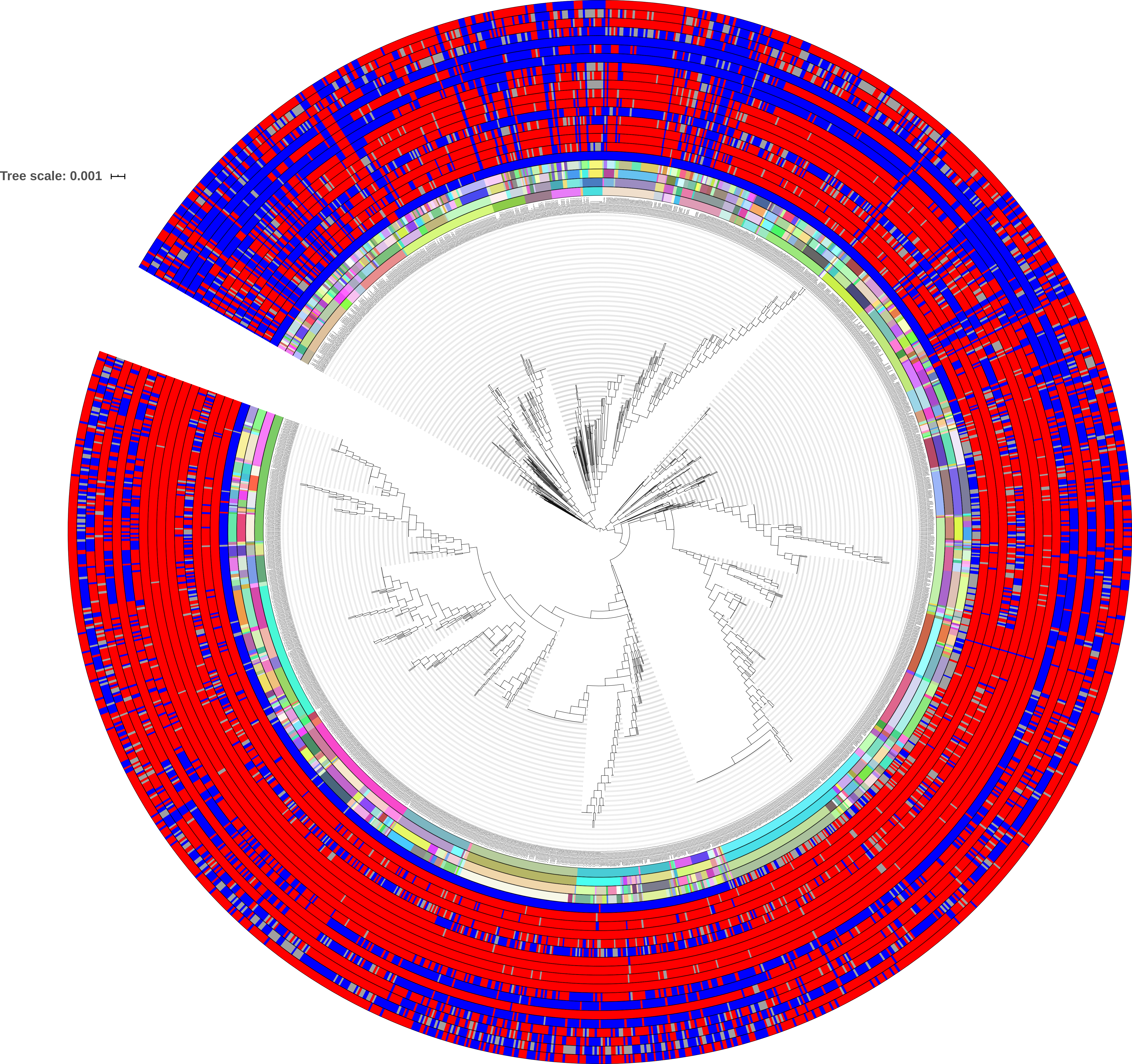

Tree scale: 0.001

Figure E. Phylogenetic tree for *Klebsiella pneumoniae* genomes. The tree was built from a concatenated alignment of 100 core genes that were not used in models. The first four rings from inside to outside depict subtrees at varying tree distances. These rings represent 97, 203, 382, and 533 clades respectively. The color depicting each subtree was chosen randomly. The remaining rings from inside to outside depict AMR phenotypes for amikacin, ampicillin/sulbactam, aztreonam, cefazolin, cefepime, cefoxitin, ceftazidime, ceftriaxone, cefuroxime sodium, ciprofloxacin, gentamicin, imipenem, levofloxacin, meropenem, piperacillin/tazobactam, tetracycline, tobramycin, and trimethoprim/sulfamethoxazole. Red represents resistance, blue represents susceptibility, and gray represents no data.

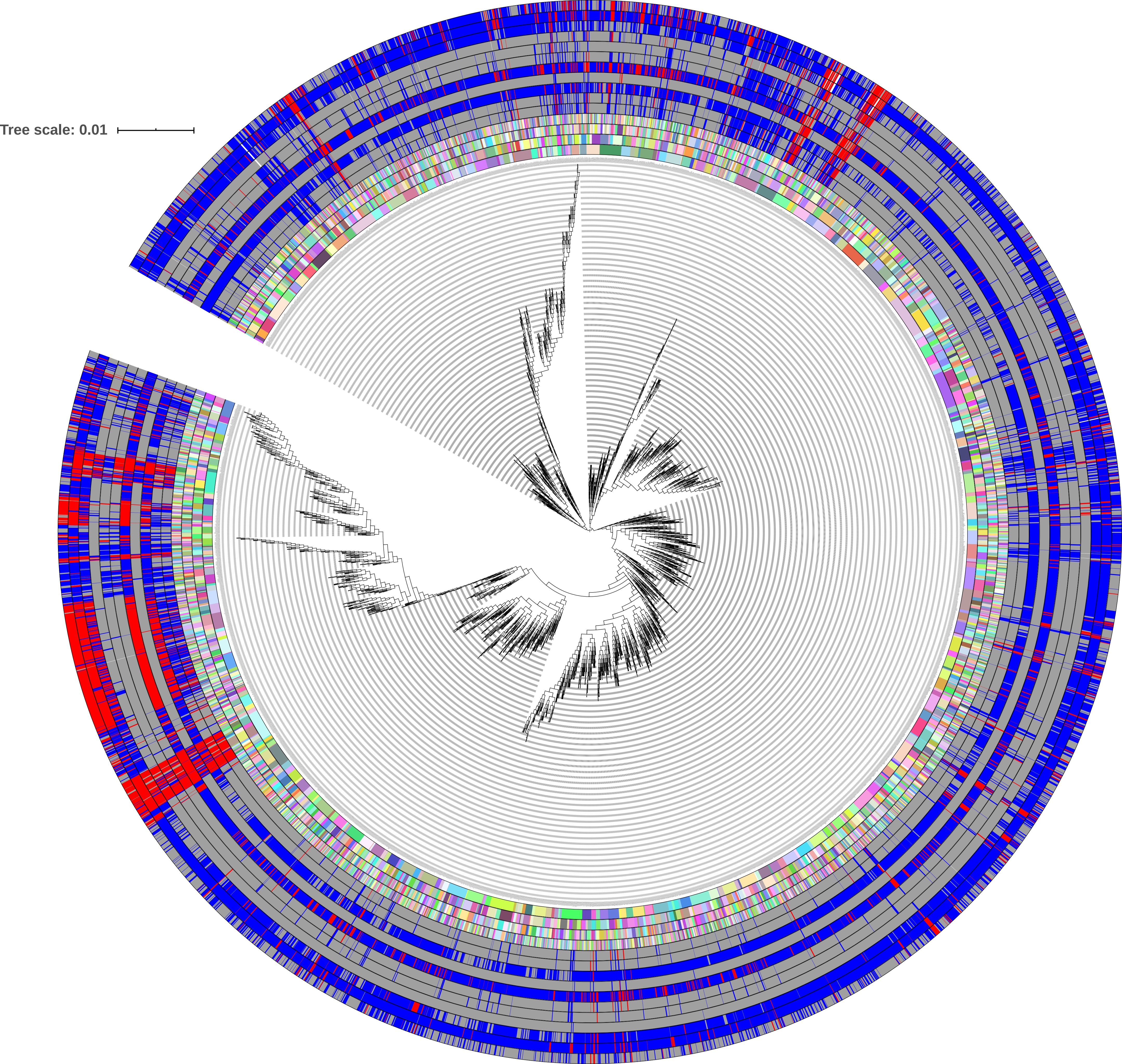
Tree scale: 0.01

Figure F. Phylogenetic tree for *Mycobacterium tuberculosis* genomes. The tree was built from a concatenated alignment of 100 core genes that were not used in models. The first four rings from inside to outside depict subtrees at varying tree distances. These rings represent 510, 1017, 1627, and 3001 clades respectively. The color depicting each subtree was chosen randomly. The remaining rings from inside to outside depict AMR phenotypes for amikacin, capreomycin, ethambutol, ethionamide, isoniazid, kanamycin, moxifloxacin, ofloxacin, pyrazinamide, rifampin, and streptomycin. Red represents resistance, blue represents susceptibility, and gray represents no data.

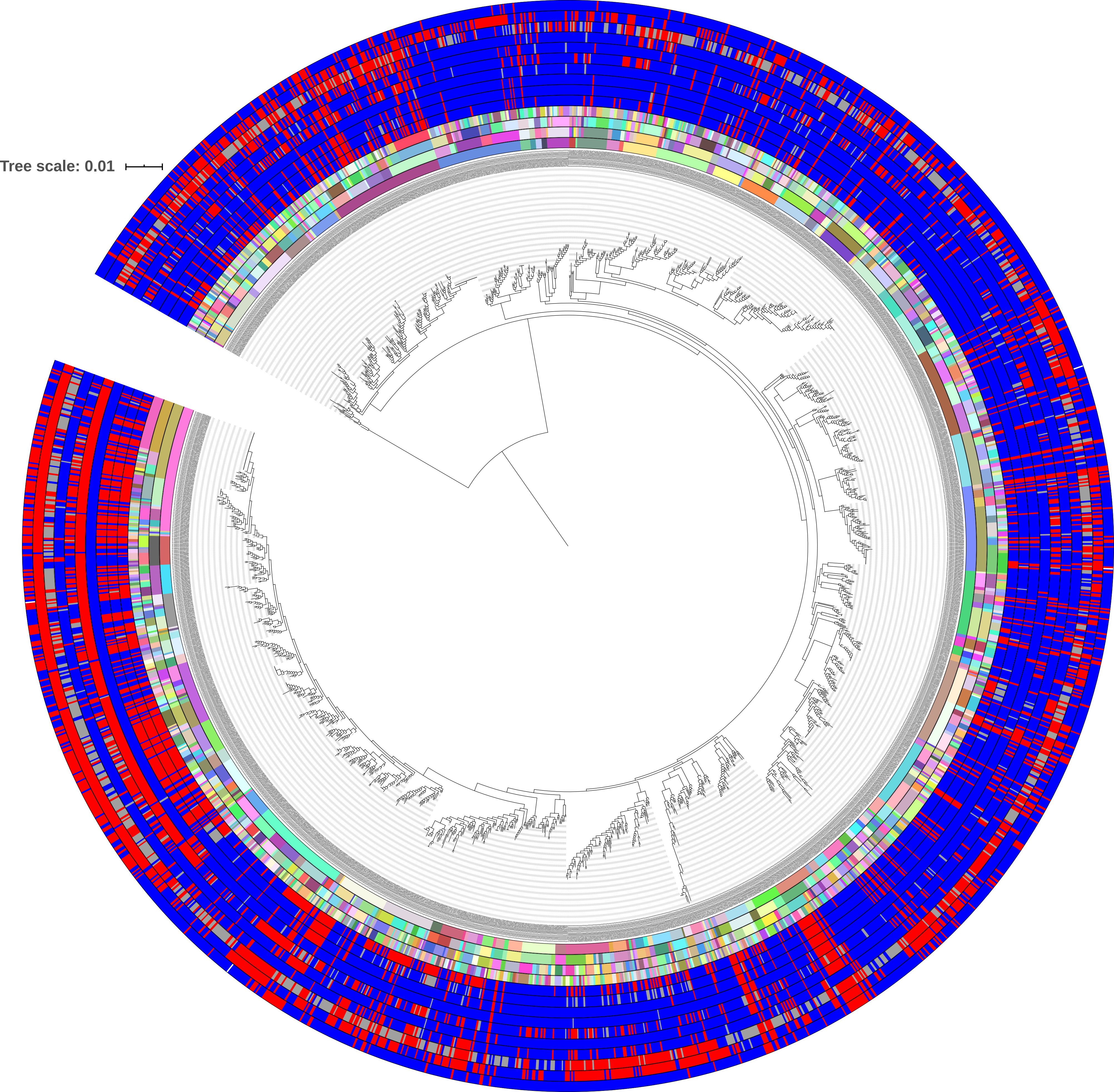

Tree scale: 0.01

Figure G. Phylogenetic tree for *Salmonella enterica* genomes. The tree was built from a concatenated alignment of 100 core genes that were not used in models. The first four rings from inside to outside depict subtrees at varying tree distances. These rings represent 141, 320, 547, and 1009 clades respectively. The color depicting each subtree was chosen randomly. The remaining rings from inside to outside depict AMR phenotypes for ciprofloxacin, clindamycin, erythromycin, fusidic acid, methicillin, and penicillin. Red represents resistance, blue represents susceptibility, and gray represents no data.

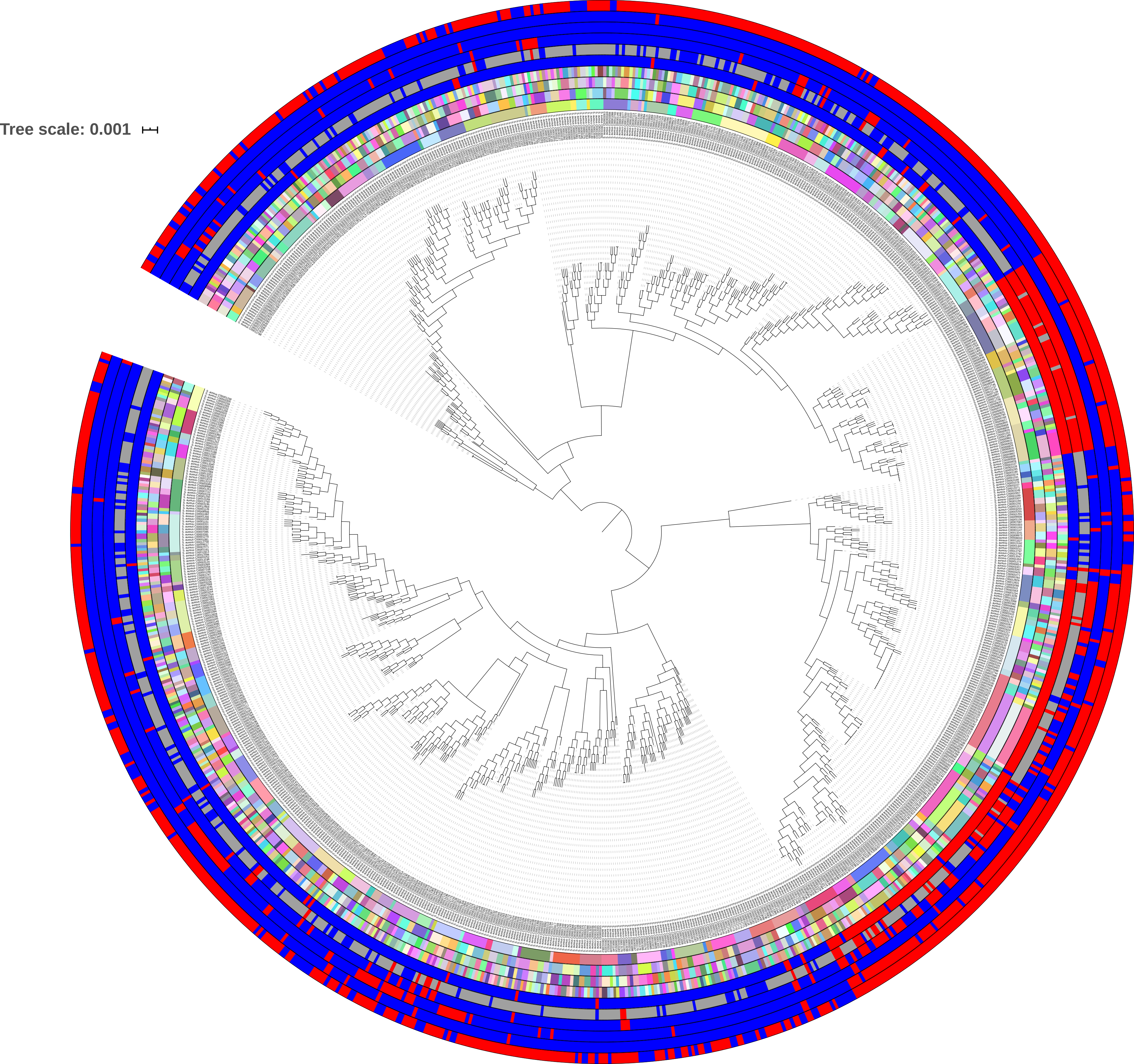
Tree scale: 0.001

Figure H. Phylogenetic tree for *Staphylococcus aureus* genomes. The tree was built from a concatenated alignment of 100 core genes that were not used in models. The first four rings from inside to outside depict subtrees at varying tree distances. These rings represent 179, 372, 509, and 802 clades respectively. The color depicting each subtree was chosen randomly. The remaining rings from inside to outside depict AMR phenotypes for ampicillin, amoxicillin-clavulanate, ceftriaxone, chloramphenicol, sulfisoxazole, cefoxitin, gentamicin, streptomycin, tetracycline, and ceftiofur. Red represents resistance, blue represents susceptibility, and gray represents no data.

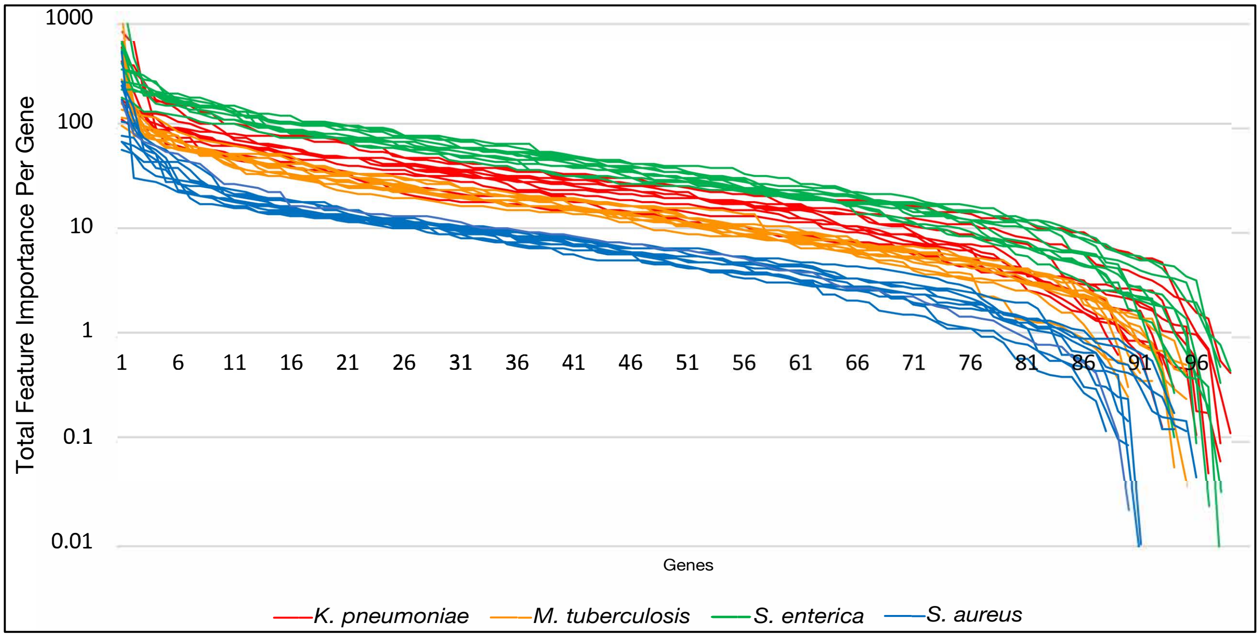

Figure I. Total feature importance per gene. K-mer-based XGB models were built for 10 non-overlapping sets of 100 core genes. The X-axis depicts each of the 100 genes sorted by total feature importance and the Y-axis is the total feature importance score. Each line depicts one model for *K. pneumoniae* (red), *M. tuberculosis* (orange), *S. enterica* (green), and *S. aureus* (blue).
